# Supplementary material for: Vitamin D Receptor Protects against Radiation-Induced Intestinal Injury in Mice via Inhibition of Intestinal Crypt Stem/Progenitor Cell Apoptosis
Source: Nutrients. 2021 Aug 24;13(9):2910. doi: 10.3390/nu13092910 (PMC8466099; doi:10.3390/nu13092910)
Supplement: Supplementary file 1 [file nutrients-13-02910-s001.zip › nutrients-1295035-supplementary.pdf]

Table S1 Composition of the rescue diet as specified by manufacturer.

Component (g/kg)	Weight (g)
Casein	200
L-Cystine	3
Lactose	200
Cellulose	50
Soybean oil	70
Vitamin and trace element premix	10
Calcium carbonate	18
Calcium phosphate	44
Starch	193
Maltodextrin	112
Sucrose	100

Table S2 siRNA sequences.

**Name**	**Sense** (5'-3')	**Antisense** (5'-3')
si-Pmaip1-1	CAGGAAGAUUGGAGAUAAA	UUUAUCUCCAAUCUUCCUG
si-Pmaip1-2	CGAAAGAGCACGAUGAGAA	UUCUCAUCGUGCUCUUUCG
si-Pmaip1-3	CCGGAGAAUUGGAGACAAA	UUUGUCUCCAAUUCUCCGG

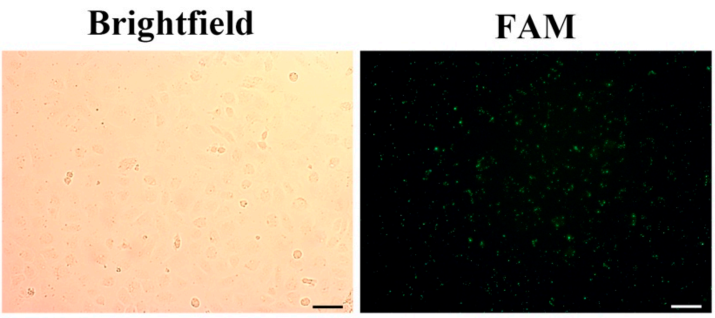

Figure S1. Transfection efficiency in IEC-6 cells determined by fluorescence microscope. The representative image shows the FAM fluorescence in the same site in the brightfield. Scale bar, 20 μm.

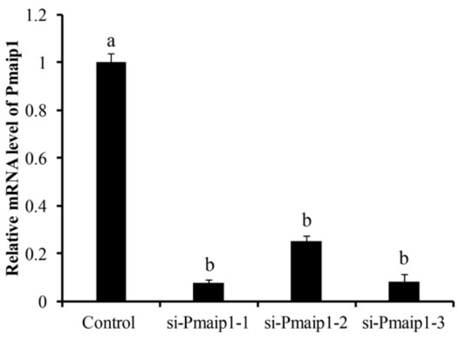

Figure S2. RT-qPCR results testing the knockdown efficiency of different pairs of si-Pmaip1 in IEC-6 cells at 24 h after si-Pmaip1 transfection. IEC-6 cells exposed to si-Pmaip1-1 and si-Pmaip1-2 had high mortality. The statistical analysis was performed by one-way ANOVA (lowercase letters indicate significant differences, $p < 0.05$).
